# Obesity Paradox in Peripheral Arterial Disease: Results of a Propensity Match Analysis from the National Inpatient Sample

**DOI:** 10.7759/cureus.4704

**Published:** 2019-05-21

**Authors:** Dipesh Ludhwani, Joyce Wu

**Affiliations:** 1 Internal Medicine, Chicago Medical School / Rosalind Franklin University of Medicine and Science, McHenry, USA; 2 Biostatistics, University of Michigan, Ann Arbor, USA

**Keywords:** national inpatient sample, obesity, obesity paradox, peripheral artery disease, propensity match, body mass index

## Abstract

Introduction

The role of obesity in cardiovascular mortality is controversial. The obesity paradox has been widely attributed to smoking in the underweight. Large-scale studies analyzing the outcomes of peripheral arterial disease (PAD) in patients with a higher body mass index (BMI) while accounting for confounders such as smoking are lacking.

Method

The 2016 National Inpatient Sample (NIS) was used to identify all admissions with a primary discharge diagnosis of PAD. A secondary diagnosis of obesity or elevated BMI was used to segregate the admissions into two groups. Propensity scores were calculated to match and control both groups for age, smoking, and diabetes, amongst other confounders. A multivariate logistic and linear regression analysis was performed to calculate the odds ratio for in-hospital mortality, amputation, need for intervention (angioplasty or bypass), acute kidney injury, hospital charges, and length of stay. Non-obesity-related PAD admissions were selected as the reference groups.

Results

Among 248,288 PAD-related admissions, 41,618 had a secondary diagnosis of obesity. After calculating propensity scores for 1-1 matching, 41,589 admissions in the PAD and obesity group were compared to a similar number of admissions in the reference population. Patients with a concomitant diagnosis of obesity had lower odds of amputation (OR=0.90, 95% CI=0.84-0.95, p<0.001), need for intervention (OR=0.66, 95% CI=0.62-0.69, p<0.0001), and in-hospital mortality (OR=0.81, 95% CI=0.74-0.87, p<0.0001). On the contrary, the odds of having acute kidney injury were higher with elevated BMI (OR=1.30, 95% CI=1.26-1.34, p<0.0001).

Conclusion

Despite increasing the risk of hypertension, diabetes, and hypertriglyceridemia, the obesity paradox continues to exist with a better short-term prognosis in patients with PAD. Future studies looking into the pathophysiology behind this phenomenon are needed.

## Introduction

Over the last few decades, obesity has emerged as a global epidemic with its prevalence constantly on the rise [1]. The role of body mass index (BMI) as a surrogate marker in defining and quantifying the degree or severity of obesity has been well-established in numerous epidemiological studies. Formally defined as a BMI of 30.0 kg/m^2^ or higher, obesity has been a significant cause of morbidity and mortality worldwide. Obesity is further stratified as class I (30 kg/m^2^ to 34.9 kg/m^2^), class II (35 kg/m^2^ to 39.9 kg/m^2^), and class III, also known as severe or morbid obesity (>40 kg/m^2^). The term ‘overweight,’ which is usually reserved for BMI between 25.0 kg/m^2^ and 29.9 kg/m^2^ is also associated with increased all-cause mortality [2-3]. The holistic impact of obesity was reflected in a report that concluded a potential decline in life expectancy due to the former’s hazardous effects on body homeostasis [4]. Obesity poses a significant health care burden to society and has been directly linked with the risks of diabetes mellitus, hypertension, dyslipidemia, cardiovascular disease, stroke, venous thrombosis, musculoskeletal complications, gastroesophageal reflux disease, and malignancy [5-7]. BMI-related mortality follows either a J-shaped or a U-shaped curve, with the lowest mortality close to a BMI of 25 kg/m2. The rates of clinical complications increase exponentially with a higher BMI [8].

Much has been studied about the pathophysiology behind obesity and its consequences. Impaired insulin signaling and the downregulation of major insulin-responsive glucose transporters are central to insulin resistance in the obese population. Vascular dysfunction resulting from adipocyte-mediated cytokine release has been implicated in triggering hypertension and dyslipidemia [9]. Despite the strong linkage between elevated BMI and cardiovascular risk factors, numerous reports have suggested favorable cardiovascular outcomes in obese patients as compared to those with normal BMI, which is otherwise referred to as the ‘obesity paradox’ [10-12]. Different theories, such as body composition, improved cardiorespiratory fitness, greater mobilization of endothelial progenitor cells, decreased thromboxane production, and increased ghrelin sensitivity, have been postulated to explain the improved prognosis in this population. Similar paradoxical results were noted in patients with peripheral arterial disease (PAD); however, this was attributed to excess mortality in the underweight who had a more pronounced history of smoking and obstructive lung disease [13]. Studies analyzing the influence of obesity in PAD outcomes in two equally matched populations while accounting for various confounders are lacking. We conducted a retrospective study to verify the existence of the obesity paradox in patients with PAD in two equally matched cohorts.

## Materials and methods

Data sources

The 2016 National Inpatient Sample (NIS), the largest all-payer database of inpatient hospitalization, was used for the study [14]. Initially founded as a component of the Health Care Utilization Project (HCUP) sponsored by the Agency for Healthcare Research and Quality (AHRQ), the NIS is released for public use each year based on the billing data submitted by hospitals to statewide data organizations across the United States (U.S.). Data is collected from 47 states covering almost 97% of the U.S. population. The NIS approximates a 20% stratified sample of discharges from all the participating hospitals, comprising over seven million annual hospitalizations. In comparison to the older versions of NIS (before the year 2012), instead of constructing samples from selected hospitals in the US, the newer NIS datasets were created utilizing 20% stratified discharges from all the hospitals in the country. The information of all available hospitalizations is available as deidentified patient demographics, discharge diagnosis as identified by the international classification of diseases, tenth revision clinical modification (ICD-10 CM), procedures performed (ICD-10 PCS), comorbid conditions, discharge status, total charges, and ultimate stay outcome. The dataset allows the identification of one primary discharge diagnosis, and up to 24 secondary discharge diagnoses or comorbidities are available for each hospital stay. The self-weighting design of the newer 2016 model provides a more accurate national estimate compared to the older versions. Since the dataset includes de-identified patient information, permission from the institutional review board was not sought.

Study population and outcomes

ICD-10-CM codes were used to identify all hospitalizations with a primary discharge diagnosis of PAD. This included patients hospitalized with stable atherosclerosis, chronic ischemia, or critical limb ischemia. All PAD-related admissions were stratified into two groups based on the presence of a secondary diagnosis code: obesity or elevated BMI or both. As discussed above, since multiple studies have shown a poor prognosis in both overweight and obese populations, we used BMI>25 kg/m^2^ as the cut-off to test for the ‘obesity paradox.’ Two groups comprising PAD with obesity or BMI>25 kg/m^2^ and PAD with BMI<25 kg/m^2^ were compared to study the primary outcome of in-hospital mortality. Secondary outcomes included the need for intervention, rates of amputation, acute kidney injury, post-catheterization complications, length of stay, and total hospital charges. We defined the need for intervention as those requiring angioplasty (balloon or stent placement) or bypass surgery. PAD-related hospitalization with BMI<25 kg/m^2^ served as the reference group.

Statistical analysis

To control for imbalances in the patient and institutional characteristics and mitigate the selection bias, we used a propensity scoring method to establish a matched sample. A propensity score was based on a multivariate logistic regression model that examined the impact of the variables listed in Table 1. Patients with similar propensity scores in the two groups (PAD subjects with and without obesity) were matched using a 1-to-1 scheme without replacement, using an eight to one digit match. All patients were 18 or older. Outcomes were compared between propensity score-matched subjects (PAD subjects with and without obesity). The paired t-test was used for comparisons of continuous variables and the McNemar test for categorical variables in the matched cohorts. All statistical analyses were performed using SAS 9.4 software (SAS Institute Inc., Cary, NC, US).

**Table 1:** Comparison of demographics and comorbidities in patients with peripheral arterial disease with and without obesity (before propensity match). SD=standard deviation

Variables	With Obesity (n=41618)	Without Obesity (n=206670)	Total (n=248288)	P-Value
	Mean (SD)	Mean (SD)	Mean (SD)	
Age, mean ± SD (years)	66.8 (11.9)	72.3 (11.9)	71.4 (11.9)	<0.0001
	N (%)	N (%)	N (%)	
Gender				
Female	19535 (46.9%)	87111 (42.1%)	106646 (43.0%)	<0.0001
Male	22058 (53.0%)	119486 (57.8%)	141544 (57.0%)	
Race				
White	30459 (73.2%)	153310 (74.2%)	183769 (74.0%)	<0.0001
Black	6814 (16.4%)	30479 (14.7%)	37293 (15.0%)	
Hispanic	3176 (7.6%)	14972 (7.2%)	18148 (7.3%)	
Asian or Pacific Islander	297 (0.7%)	2747 (1.3%)	3044 (1.2%)	
Native American	206 (0.5%)	1026 (0.5%)	1232 (0.5%)	
Other	666 (1.6%)	4130 (2.0%)	4796 (1.9%)	
Household income				
$1-24,999	14320 (34.4%)	68585 (33.2%)	82905 (33.4%)	<0.0001
$25,000-34,999	11492 (27.6%)	54679 (26.5%)	66171 (26.7%)	
$35,000-44,999	9490 (22.8%)	47880 (23.2%)	57370 (23.1%)	
45,000 or more	6312 (15.2%)	35511 (17.2%)	41823 (16.8%)	
Comorbidities	N (%)	N (%)	N (%)	
Smoking	6962 (16.7%)	44201 (21.4%)	51163 (20.6%)	<0.0001
Coronary Artery Disease	23025 (55.3%)	115902 (56.1%)	138927 (56.0%)	0.0046
Congestive Heart Failure	5527 (13.3%)	23017 (11.1%)	28544 (11.5%)	<0.0001
Chronic Kidney Disease	18643 (44.8%)	78386 (37.9%)	97029 (39.1%)	<0.0001
Diabetes Mellitus	27769 (66.7%)	92925 (45.0%)	120694 (48.6%)	<0.0001
Hypertension	18038 (43.3%)	97601 (47.2%)	115639 (46.6%)	<0.0001

## Results

A total of 248,228 admissions with a primary discharge diagnosis of PAD were identified, of which 41,618 (16.76%) had a secondary discharge diagnosis of obesity or BMI greater than 25 kg/m^2^. Table 1 demonstrates the baseline characteristics of both groups before matching propensity scores. The mean age of patients in the obesity group was lower as compared to non-obese patients (66.8 vs. 72.3, p<0.0001). As was pointed out in previous studies, the rates of smoking were higher in patients with BMI<25 kg/m^2^ when compared to patients with BMI>25 kg/m^2^ (21.4% vs. 16.7%, p<0.0001). Higher BMI was also associated with concomitant secondary diagnosis of congestive heart failure (13.3% vs 11.1%, p<0.0001), chronic kidney disease (44.8% vs 37.9%, p<0.0001), and diabetes mellitus (66.7% vs 45.0%, p<0.0001).

After calculating propensity scores; matching both cohorts and accounting for missing information, we compared 41,589 (16.75%) PAD admissions with elevated BMI (BMI>25 kg/m^2^) or secondary diagnosis of obesity to a similar number of hospitalizations in the reference group. In the obesity group, 4356 (10.47%) had BMI between 25 to 29.9 kg/m^2^, 7195 (17.30%) had BMI between 30.0 to 34.9 kg/m^2^, 9505 (22.85%) had BMI between 35.0 to 39.9 kg/m^2^, and 12522 (30.11%) admissions had BMI>40 kg/m^2^. A total of 8011 admissions (19.26%) had secondary discharge diagnosis of overweight, obesity, or morbid obesity; however, the BMI range was not specified. Table 2 compares baseline characteristics between both groups after calculating propensity scores. After propensity matching, the standardized mean difference between both groups was less than 5%. The mean age of the cohort after matching was 66.9±11.7 years. In the obesity group, 53% were males and 46% were females as compared to 54.6% males and 45.4% females in the non-obese group. The pre-match confounding effect of smoking was negated post-match, and the rates for both groups were 16.7% vs. 17.0%.

**Table 2:** Comparison of demographics and comorbidities in patients with peripheral arterial disease after calculating propensity scores. SD=standard deviation, NA=not available.

Variables	With Obesity (n=41589)	Without Obesity(n=41589)	Total (n=83178)	Standardized mean Differences
	Mean (SD)	Mean (SD)	Mean (SD)	
Age, mean ± SD (years)	66.8 (11.9)	66.9 (11.5)	66.9 (11.7)	-0.12
	N (%)	N (%)	N (%)	
Gender				
Female	19533 (47.0%)	18861 (45.4%)	38394 (46.2%)	3.2
Male	22056 (53.0%)	22728 (54.6%)	44784 (53.8%)	
Race				
White	30442 (73.2%)	30591 (73.6%)	61033 (73.4%)	-0.8
Black	6803 (16.4%)	6884 (16.6%)	13687 (16.5%)	
Hispanic	3175 (7.6%)	3119 (7.5%)	6294 (7.6%)	
Asian or Pacific Islander	297 (0.7%)	327 (0.8%)	624 (0.8%)	
Native American	206 (0.5%)	163 (0.4%)	369 (0.4%)	
Other	666 (1.6%)	505 (1.2%)	1171 (1.4%)	
Household income				
$1-24,999	14308 (34.4%)	14691 (35.3%)	28999 (34.9%)	-1.6
$25,000-34,999	11487 (27.6%)	11025 (26.5%)	22512 (27.1%)	
$35,000-44,999	9485 (22.8%)	9333 (22.4%)	18818 (22.6%)	
45,000 or more	6309 (15.2%)	6540 (15.7%)	12849 (15.4%)	
Comorbidities	N (%)	N (%)	N (%)	
Smoking	6957 (16.7%)	7070 (17.0%)	14027 (16.9%)	-1.5
Coronary Artery Disease	23011 (55.3%)	23610 (56.8%)	46621 (56.0%)	-2.9
Congestive Heart Failure	5526 (13.3%)	4679 (11.3%)	10205 (12.3%)	2.6
Chronic Kidney Disease	18627 (44.8%)	18384 (44.2%)	37011 (44.5%)	1.3
Diabetes Mellitus	27751 (66.7%)	28281 (68.0%)	56032 (67.4%)	-3.1
Hypertension	18026 (43.3%)	17491 (42.1%)	35517 (42.7%)	1.7
Propensity Scores	0.03126	0.03126	NA	0

**Figure 1:**
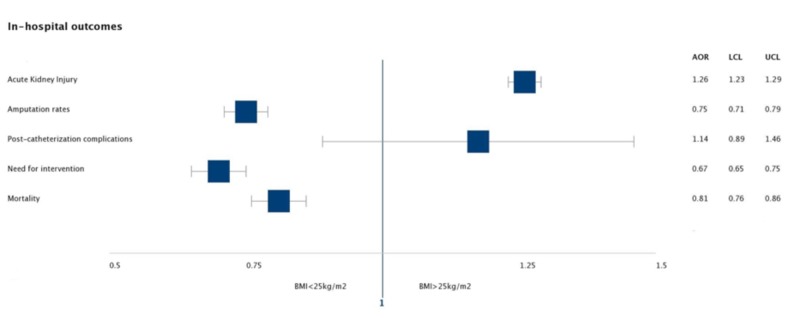
Calculated adjusted odds ratio for various in-hospital outcomes before calculating propensity scores. BMI=Body mass index, AOR=adjusted odds ratio, LCL=lower limit of confidence interval, UCL=Upper limit of confidence interval

Before matching both cohorts, the rates of amputation (5.03% vs 5.0%, p<0.0001), need for intervention (8.6% vs 6.4%, p<0.0001), and in-hospital mortality (3.7% vs 2.7%, p<0.0001) were lower in PAD hospitalizations with a higher BMI. The rates of acute kidney injury (22.7% vs 27.4%, p-value<0.0001), and post-catheterization complications (0.2% vs 0.2%, p=0.30074) were higher in the obesity group, however, the post-catheterization complication rates were statistically not significant (Figure 1).

**Figure 2:**
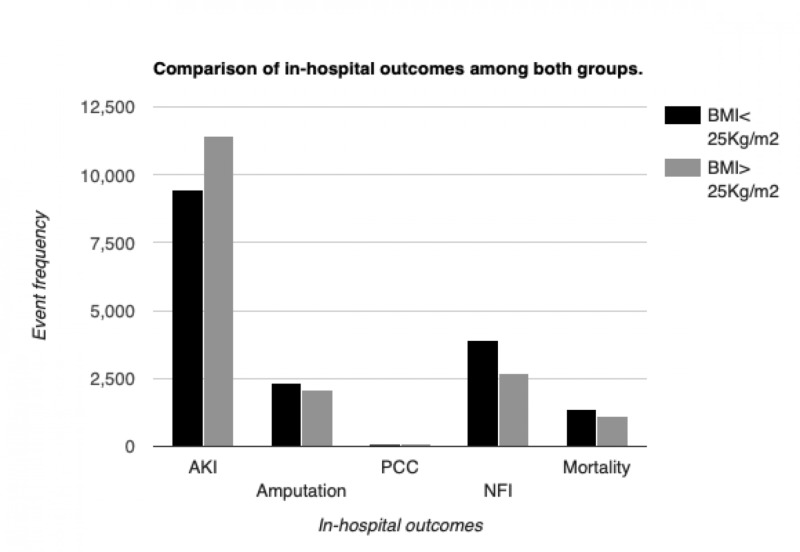
Comparison of in-hospital outcomes among both groups after calculating propensity scores. Except PCC, all differences were statistically significant. PCC=Post-catheterization complications, NFI=Need for intervention

**Figure 3:**
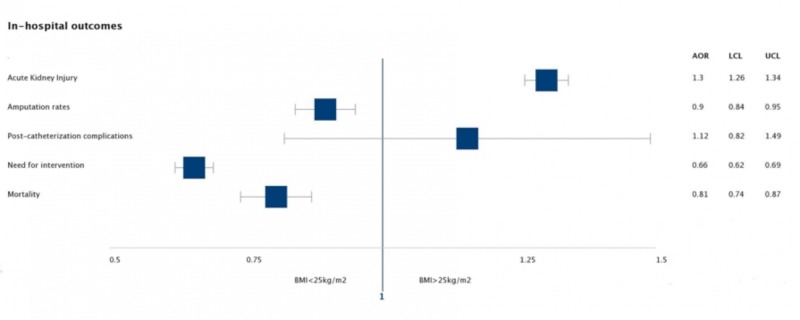
Calculated adjusted odds ratio for various in-hospital outcomes after calculating propensity scores. BMI=Body mass index, AOR=adjusted odds ratio, LCL=lower limit of confidence interval, UCL=Upper limit of confidence interval

Post-matching, lower BMI (<25 kg/m^2^) was associated with higher rates of in-hospital mortality (3.30% vs 2.68%, p<0.0001), amputation (5.66% vs 5.30%, p<0.0001), and need for intervention (9.44% vs 6.42%, p<0.0001). On the contrary, patients with higher BMI (>25 kg/m^2^) had increased rates of acute kidney injury (AKI) compared to the reference population (27.43% vs. 22.77%, p<0.0001) (Figures 2-3). All hospitalizations with BMI>25 had a higher mean length of stay (6.5 days vs. 5.8 days, p<0.0001) and total hospital charges ($73,046.17 vs. $66,540.94, p<0.0001).

## Discussion

The overall prevalence of PAD increases substantially from 3%-10% to 15%-20% after the age of 70years [15]. The prognosis of PAD is significantly worse in diabetes, end-stage renal disease, and active smokers. In our retrospective study, we observed an inverse relationship between obesity and mortality in patients with PAD, consistent with the findings of the ‘obesity paradox,’ as described previously in the literature. The results in previous studies, however, were not reproducible once adjusted for smoking and COPD [13]. To the best of our knowledge, this is the largest observational study where one to one matching was performed, and findings were tested before and after the application of propensity scores. In our study, the presence of a higher BMI independently decreased the odds of in-hospital mortality by 19% in patients with PAD. Uretsky et al. studied the long-term outcomes of obesity in patients with hypertension and found a 30% lower all-cause mortality in overweight and obese population despite less effective blood pressure control in this population [16].

A similar contradictory role of obesity has also been reported with heart failure. Obesity increases cardiac output, cardiac workload, and filling pressures, cumulating increasing the risk of heart failure. Despite the known adverse effects of the obese state on cardiovascular hemodynamics, the overall prognosis tends to be superior in this population [17-18]. A meta-analysis conducted by Oreopoulus et al. showed a 19% and 40% cardiovascular mortality benefit in the overweight and obese populations, respectively [19]. The exact pathophysiology behind this phenomenon remains elusive. Excess adipose tissue produces soluble tumor necrosis factor receptor, which plays a protective role by neutralizing the toxic effects of tumor necrosis factor-alpha [20]. Higher metabolic reserve and attenuation of the sympathetic and renin-angiotensin systems all seem to favor better outcomes in the obese population.

Nehler MR et al. reported the mean annualized prevalence of critical limb ischemia (CLI) around 1.33%. The same study estimated the progression of PAD to CLI in 11.08% of cases [21]. The prevalence seems to be higher in diabetics; however, similar studies based on BMI are lacking [22]. In our study, the prevalence of CLI was higher in patients with BMI<25 kg/m^2^. The prevalence of CLI in the propensity-matched cohort was 7.93% in patients with BMI<25 kg/m^2^ as compared to 5.17% in those with higher BMI. This raises the possibility of CLI being one of the driving factors associated with poorer PAD outcomes in patients with low to normal BMI. CLI, the most severe presentation of PAD, is characterized by multisegmental blood flow impairment with ischemic rest pain, ulceration, or gangrenous formation. CLI is associated with high amputation rates and worse mortality outcomes, especially in elderly and diabetic patients [23]. The initial mortality rate of 20% within six months of diagnosis can rise to 50% at the end of five years [23]. The amputation rates for CLI is estimated to be somewhere between 10% and 40% at six months [24].

The underlying pathophysiology behind coronary artery disease and PAD include atherosclerotic plaque formation, resulting in compromised blood flow. Traditional cardiovascular risk factors, such as age, smoking, hypertension, and obesity, have all been implicated in the progression of both conditions [25]. Despite this, several studies have shown the obesity survival paradox, highlighting an association between higher BMI and survival [10-12] The role of obesity in PAD outcomes is also not well-established. While some studies have shown clear survival benefits [13,26], other studies have failed to show similar results [26]. Various theories explaining this phenomenon have been cited in the literature. Obese patients are more likely to seek medical care at an earlier stage given other comorbid conditions potentially influencing long-term outcomes, more aggressive treatment, and medication compliance in this population. High suspicion and better screening have reduced the time to diagnosis drastically. The presence of occult malignancy and COPD have also been cited as potential explanations for poor outcomes in the underweight [13]. Obesity serves as a nutritional reserve against cardiac cachexia, which is particularly important in older patients, as it carries a poor prognosis. The detrimental effects of obesity have also been attributed to concomitant inflammation, as favorable outcomes have been noted in the obese, with low levels of the inflammatory marker; while, on the other hand, obesity with elevated inflammatory markers tends to have worse consequences [27].

More recently, the role of BMI as an assessment tool for obesity or cardiovascular risk factor has come under scrutiny [28]. The limitation of BMI in differentiating excess body fat from lean body mass has been well-established [29]. Better outcomes in patients with a higher BMI but a predominant lean body mass has also been attributed as a contributing factor explaining the obesity paradox. Studies using the abdominal circumference and waist-to-hip ratio have gained attention due to the above-mentioned limitations of BMI. Studies using fat percentage along with BMI have also shown similar paradoxical results [30]. Despite this, purposeful weight loss has been associated with better exercise capacity, inflammation, and lipid profile [30]. Based on our results, a higher BMI is associated with a better prognosis after adjusting for age, sex, and other comorbidities. Despite the paradox, the author recommends purposeful long-term weight loss to improve obesity-related morbidity.

The following limitations should be considered when interpreting the findings of our study. First, since the NIS database relies heavily on ICD-10-CM diagnosis codes, any error in coding can affect the validity of the study. Second, we were unable to assess the medical compliance of the patients leading to the hospitalization. The comparison of obese patients being medically more compliant or vice versa compared to those with low or normal BMI could not be established. We were unable to clarify whether those with ICD codes for tobacco use were active or former smokers and, if so, the extent of smoking was not quantified. We lacked data on out-of-hospital long-term outcomes in these patients. The present study also had its strength in being the largest retrospective observational study testing obesity paradox in propensity-matched cohorts accounting for numerous confounders among patients admitted with PAD.

## Conclusions

We found the prognosis of PAD was inversely proportional to the BMI status of the patient. Various theories have been postulated explaining this phenomenon and questioning the utility of BMI as an indicator of body fat. Future studies looking further into the pathophysiology and consistency of these results with other obesity indices are needed.
